# Development, Safety, and Therapeutic Evaluation of Voriconazole-Loaded Zein–Pectin–Hyaluronic Acid Nanoparticles Using Alternative In Vivo Models for Efficacy and Toxicity

**DOI:** 10.3390/pharmaceutics17020231

**Published:** 2025-02-11

**Authors:** Margani Taise Fin, Kelvin Sousa dos Santos, Marcos William de Lima Gualque, Rafaela Cristine dos Santos, Natália Cristina Morici Aoki, Marcos Ereno Auler, Ana Marisa Fusco-Almeida, Maria José Soares Mendes-Gianinni, Rubiana Mara Mainardes

**Affiliations:** 1 Laboratory of Nanostructured Formulations, Universidade Estadual do Centro-Oeste (UNICENTRO), Alameda Élio Antônio Dalla Vecchia, 838, Guarapuava 85040-167, PR, Brazil; margani.fin@gmail.com; 2 Department of Clinical Analysis, School of Pharmaceutical Sciences, Universidade Estadual Paulista Júlio de Mesquita Filho (UNESP), Rodovia Araraquara Jaú, Km 01, Araraquara 14801-902, SP, Brazil; k.santos@unesp.br (K.S.d.S.); mw.gualque@unesp.br (M.W.d.L.G.); rafaela.cristine@unesp.br (R.C.d.S.); natalia.aoki@unesp.br (N.C.M.A.); ana.marisa@unesp.br (A.M.F.-A.); maria.giannini@unesp.br (M.J.S.M.-G.); 3 Pharmacy Department, Universidade Estadual do Centro-Oeste (UNICENTRO), Alameda Élio Antônio Dalla Vecchia, 838, Guarapuava 85040-167, PR, Brazil; aulerms@gmail.com

**Keywords:** voriconazole, nanoparticles, *Candida* infections, *Caenorhabditis elegans*, *Galleria mellonella*, antifungal therapy

## Abstract

**Background/Objectives**: Fungal infections caused by *Candida* species remain a significant clinical challenge, exacerbated by limitations in current antifungal therapies, including toxicity and poor bioavailability. This study aimed to develop and evaluate voriconazole-loaded zein–pectin–hyaluronic acid nanoparticles (ZPHA-VRC NPs) as a novel drug delivery system to enhance efficacy and reduce toxicity. Alternative in vitro and in vivo models were utilized to assess the safety and therapeutic potential of the nanoparticles. **Methods**: ZPHA-VRC NPs were prepared using a nanoprecipitation method and characterized for particle size, polydispersity index, zeta potential, and encapsulation efficiency. Antifungal activity was assessed via MIC assays against *Candida albicans*, *C. krusei*, and *C. parapsilosis*. Cytotoxicity was evaluated on Vero cells, while in vivo toxicity and efficacy were assessed using *Galleria mellonella* and *Caenorhabditis elegans* models. The therapeutic efficacy was further evaluated in an infected *Caenorhabditis elegans* model using survival and health scores. **Results**: ZPHA-VRC nanoparticles exhibited favorable physicochemical properties, including a particle size of approximately 192 nm, a polydispersity index of 0.079, a zeta potential of −24 mV, and an encapsulation efficiency of 34%. The nanoparticles retained antifungal activity comparable to free voriconazole while significantly reducing cytotoxicity. In vivo studies using *G. mellonella* and *C. elegans* demonstrated that ZPHA-VRC NPs markedly improved survival rates, reduced fungal burden, and enhanced health scores in infected models, outperforming the free drug. Additionally, the nanoparticles exhibited a superior safety profile, minimizing systemic toxicity while maintaining therapeutic efficacy. **Conclusions**: ZPHA-VRC NPs offer a safer and more effective delivery system for VRC, addressing the limitations of conventional formulations. The integration of alternative efficacy and safety models highlights their value in preclinical research.

## 1. Introduction

Fungal infections caused by *Candida albicans* remain a pressing global health concern, particularly in immunocompromised populations, including individuals with HIV/AIDS, cancer patients undergoing chemotherapy, and organ transplant recipients receiving immunosuppressants. These infections, ranging from superficial mucosal conditions to life-threatening systemic diseases, pose significant challenges for both patients and healthcare systems. Systemic *Candida* infections are associated with alarmingly high mortality rates, exceeding 40% in severe cases, even with available antifungal treatments, and contribute to over 400,000 cases annually worldwide [1]. This species is the most prevalent, responsible for approximately 70% of fungal infections globally, and remains a primary agent of invasive infections in hospital settings. The widespread use of indwelling medical devices, such as catheters and implants, exacerbates the problem by facilitating colonization and biofilm formation, further complicating treatment and increasing patient morbidity [2].

The pharmacological arsenal against *Candida* infections includes polyenes, echinocandins, triazoles, and flucytosine, with echinocandins being the first-line treatment for invasive candidiasis. However, triazoles like voriconazole (VRC) remain critical due to their broad-spectrum activity, efficacy against *C. albicans* and other pathogenic fungi, and oral bioavailability [3,4]. Despite its clinical importance, voriconazole’s therapeutic potential is limited by significant challenges, including poor aqueous solubility, dose-dependent hepatotoxicity, phototoxicity, and non-linear pharmacokinetics caused by saturable metabolism [5,6,7]. These limitations underscore the urgent need for innovative drug delivery systems capable of enhancing voriconazole’s efficacy, bioavailability, and safety profile.

A major challenge in antifungal therapy is the rapid emergence of resistance and the high toxicity associated with prolonged drug use. The extensive utilization of antifungals not only drives resistance but also leads to cumulative toxic effects that can have long-term consequences for patients [8]. To address these issues, research has focused on developing novel antifungal delivery systems with improved bioavailability, reduced toxicity, and alternative mechanisms of action. These agents are particularly relevant for combination therapy strategies, which aim to enhance treatment efficacy while mitigating the risk of resistance development. Furthermore, advanced drug delivery systems, including nanoparticle-based formulations, offer a promising approach to overcoming the limitations of conventional therapies by improving drug stability, controlled release, and targeted delivery, ultimately optimizing antifungal treatment outcomes [9,10,11,12]. 

Nanoparticles based on natural vegetable polymers have shown remarkable promise in this field [13,14,15]. Zein, a hydrophobic maize-derived protein, is widely recognized for its ability to self-assemble into stable nanoparticles, encapsulating hydrophobic drugs while protecting them from degradation [16,17]. It possesses notable characteristics, including biodegradability, biocompatibility, high molecular binding affinity, and efficient cellular internalization, while also exhibiting amphiphilic behavior [18]. These properties confer broad applicability in advanced drug delivery systems, as well as the controlled release of bioactive compounds like essential oils, antimicrobial agents, and antitumor drugs. Additionally, zein nanoparticles demonstrate the capacity to protect encapsulated compounds from adverse gastric conditions, further enhancing their potential in pharmaceutical formulations [19].

Hyaluronic acid, a biocompatible polysaccharide with mucoadhesive properties, enhances the retention of nanoparticles at mucosal surfaces and facilitates targeted drug delivery [7,20]. Pectin, a natural polysaccharide commonly used as a stabilizing agent, further contributes to the structural integrity and stability of zein-based nanoparticles by reducing aggregation and improving dispersibility [7,21]. Together, these biopolymers create a synergistic platform capable of improving the solubility, stability, and controlled release of poorly soluble drugs like voriconazole while minimizing systemic toxicity and improving efficacy.

Parallel to the advancements in nanotechnology, alternative models for assessing drug safety and efficacy have gained prominence. Traditional vertebrate models, while valuable, are associated with ethical concerns, higher costs, and regulatory challenges. In this context, in vivo models such as *Galleria mellonella* and *Caenorhabditis elegans* have emerged as reliable, cost-effective, and ethically sound alternatives. *Galleria mellonella*, a larvae model, offers unique advantages due to its conserved innate immune system, which includes hemocytes and signaling pathways analogous to those found in mammals. This model has proven particularly valuable in evaluating host–pathogen interactions and the efficacy of antifungal agents. Similarly, *C. elegans* has emerged as a powerful model for toxicity assessments and the study of fungal pathogenicity, owing to its genetic tractability and suitability for high-throughput analyses. Furthermore, these models allow large-scale preliminary studies with high reproducibility and translational potential [22,23,24]. By integrating *G. mellonella* and *C. elegans* into this study, we aim to bridge the gap between in vitro analyses and more complex mammalian studies, providing a robust, ethical, and efficient approach to evaluating the therapeutic potential of voriconazole-loaded nanoparticles.

In a previous study, we demonstrated the ability of voriconazole-loaded zein–pectin–hyaluronic acid nanoparticles (ZHA-VRC NPs) to achieve controlled drug release and significantly enhance oral bioavailability in rats, highlighting its potential to overcome the pharmacokinetic challenges of voriconazole [25]. Building upon these findings, the current study aims to evaluate the toxicity and therapeutic efficacy of these nanoparticles through alternative in vitro and in vivo models, including Vero cell cytotoxicity assays, *Galleria mellonella*, and *Caenorhabditis elegans*. By integrating nanotechnology with ethical, alternative preclinical models, this work offers a comprehensive strategy for advancing antifungal therapy, enhancing voriconazole’s therapeutic potential, and improving patient outcomes in the fight against fungal infections.

## 2. Materials and Methods

### 2.1. Materials

Voriconazole (≥98% purity), zein, pectin from apple (50–75% esterification), dimethyl sulfoxide (DMSO), and 3-(4,5-dimethyl-2-thiazolyl)-2,5-diphenyl-2H-tetrazolium bromide (MTT) were purchased from Sigma-Aldrich^®^ (St. Louis, MO, USA). Hyaluronic acid (50–90 kDa) was purchased from Contipro (Dolní Dobrouč, Pardubice Region, Czech Republic). Isopropanol was obtained from Synth^®^ (Diadema, São Paulo, Brazil). Ultrapure water was prepared using a Millipore^®^ purification system (Burlington, MA, USA). Sabouraud Dextrose Agar was acquired from Kasvi^®^ (Curitiba, Paraná, Brazil). RPMI 1640 medium, DMEM medium modified by Eagle, and fetal bovine serum were sourced from Gibco^®^ (Grand Island, NY, USA).

### 2.2. Design of Experiment

A factorial design (2^2^) was applied to identify the critical factors influencing the preparation of zein–pectin–hyaluronic acid nanoparticles (ZPHA-VRC NPs) and their key physicochemical properties: particle size, polydispersity index, zeta potential, and entrapment efficiency. Two independent variables were evaluated at two levels (−1 and +1) with a center point (0), as summarized in Table 1. The factors were X_1_ (zein amount: 20–60 mg) and X_2_ (hyaluronic acid amount: 5–20 mg), while the voriconazole (VRC) concentration was maintained constant at 1 mg/mL with a fixed organic to aqueous phase ratio (O:A) of 1:5. The responses were particle size (nm) (R_1_), polydispersity index (R_2_), zeta potential (mV) (R_3_), and entrapment efficiency (EE%) (R_4_).

### 2.3. Preparation of ZPHA-VRC NPs

ZPHA-VRC NPs were prepared via the nanoprecipitation method. The organic phase was prepared by dissolving zein in 1 mL of 70% isopropyl alcohol (pH 5.7), followed by the addition of VRC. This solution was incubated in a shaker at 100 rpm and 25 °C for 2 h. The aqueous phase contained hyaluronic acid and pectin, adjusted to pH 4.0. Subsequently, 2 mL of the organic phase was added dropwise to 10 mL of the aqueous phase under magnetic stirring at 600 rpm for 30 min. Solvent removal was performed using SpeedVac for 45 min. The nanoparticle suspension was diluted to 10 mL with ultra-pure water. Drug-free nanoparticles (ZPHA NPs) were prepared following the same protocol, excluding VRC. Other complementary analytical methods were performed in the study by Fin et al., 2024 [25].

### 2.4. Particle Size, Polydispersity Index, and Zeta Potential of ZPHA-VRC NPs

The particle size and polydispersity index (PDI) were measured using dynamic light scattering (DLS) (BIC 90 Plus, Brookhaven, New York, NY, USA). For analysis, 10 µL of the nanoparticle suspension was diluted in 3 mL of ultrapure water and transferred to a cuvette. Measurements were conducted at a scattering angle of 90°, 25 °C, and a wavelength of 659 nm.

Zeta potential was determined by diluting 40 µL of the nanoparticle suspension in 1 mL of a 1 mM KCl solution. The sample was introduced into an electrophoretic cell under a potential of *±*150 mV, and measurements were performed using a ZetaSizer Nano ZS (Malvern, Cambridge, UK). Final values were averaged over three independent replicates.

### 2.5. Entrapment Efficiency Detemination

Entrapment efficiency (EE) was determined by measuring the amount of non-encapsulated VRC. Freshly prepared ZPHA-VRC NPs dispersions were centrifuged at 11,269× *g* for 15 min, and the supernatant was collected to quantify the free drug concentration using high-performance liquid chromatography (HPLC) (Alliance 2695, Waters, Milford, MA, USA) with photodiode array detector set at 255 nm. The mobile phase consisted of acetonitrile–water–formic acid in the ratio 60:40:0.5 (*v*/*v*/*v*) isocratically eluted at 1 mL/min. The EE was calculated using Equation (1):

(1) $$EE\%=\frac{\left[VRC\right]i-[VRC]sup}{[VRC]i}\times 100$$

where [VRC]i is the initial drug concentration and [VRC]sup is the unentrapped VRC in the supernatant. All measurements were conducted in triplicate for three independent experiments.

### 2.6. Determination of Antifungal Activity In Vitro

#### 2.6.1. Microorganism Cultivation and Inoculum Preparation

The antifungal activity was evaluated using *Candida albicans* ATCC 90028, *Pichia kudriavzevii* (formerly *Candida krusei*) (ATCC 6258), and *Candida parapsilosis* (ATCC 22019), obtained from the mycology collection at UNESP. The inoculum was prepared from colonies cultured on Sabouraud Dextrose Agar (SDA) at 35 °C for 24–48 h, then suspended in sterile saline (0.85%). Cell viability was assessed using trypan blue staining. The suspension was diluted in RPMI 1640 medium buffered with MOPS to achieve a final concentration of 5 × 10^6^ cells/mL following CLSI M27 protocol [26].

#### 2.6.2. Minimum Inhibitory Concentration (MIC) Measurements

MIC was determined using a broth microdilution assay according to CLSI guidelines, with slight modification. ZPHA-VRC NPs and free VRC were tested at concentrations ranging from 0.007 to 4.0 µg/mL and fluconazole ranging from 0.125 to 64.0 µg/mL. Negative controls included RPMI medium alone and RPMI with DMSO. The positive growth control consisted of fungal inoculum in RPMI-1640 medium. Microdilution plates were incubated at 35 °C for 24 h. Readings were confirmed spectrophotometrically at 530 and 600 nm (Synergy BioTek, Agilent, Santa Clara, CA, USA). The MIC was determined by the total inhibition of growth compared to the positive control. Three independent experiments were conducted in triplicate.

### 2.7. Cytotoxicity Assay in Vero Cells

The Vero cell line, originating from the kidney of an adult African green monkey (ATCC CCL-81), was selected based on the systemic activity of voriconazole. The cells were grown in a DMEM medium modified by Eagle, with the addition of 1% antibiotic solution and 10% fetal bovine serum. The cultures were maintained at 37 °C in a humidified incubator with 5% CO_2_ and 95% air. Consistent culture conditions and cell passage numbers were ensured across all experiments.

Cell viability was evaluated using the MTT assay. Vero cells were seeded into 96-well plates (5 × 10^4^ cells/well) and exposed to ZPHA-VRC NPs or free VRC (5–500 µg/mL) for 24, 48, and 72 h. Following exposure, cells were treated with MTT solution (500 µg/mL in DMEM) for 3 h. Post-incubation, the MTT solution was removed, and the plates were dried. To dissolve the resultant formazan crystals, 100 µL of DMSO was added to each well, and the absorbance was measured at 550 nm using a Synergy BioTek plate reader (Agilent Technologies, Santa Clara, CA, USA). Untreated cells and cells treated with DMSO (20, 10, 5, and 1%) served as negative and positive controls, respectively.

### 2.8. In Vivo Toxicity Evaluation Using Caenorhabditis elegans

The toxicity of ZPHA-VRC NPs was assessed using *C. elegans* N2 strains cultured on nematode growth medium (NGM) with *Escherichia coli* OP50 as a food source [27]. The synchronized larvae at the L3/L4 stages were washed repeatedly with 5 mL of 50 mM NaCl, totaling three to four washes. The supernatant was transferred to a 15 mL conical tube, and the volume was adjusted to 10 mL using 50 mM NaCl. The larvae were allowed to settle for 15 min. This washing process was repeated three times. In the final wash, the supernatant was discarded, leaving 500 µL of 50 mM NaCl to facilitate homogenization of the larvae for counting. For counting, 10 µL of the larval suspension was placed on a glass slide in duplicate. The total larval count per drop (10 µL) was performed.

The toxicity assay was conducted in 96-well plates, with each well containing approximately 20 larvae/100 µL. The groups tested included the negative control (culture medium only), the positive control (larvae without treatment), and the experimental groups (larvae + treatment). The plates were incubated in a humid chamber at 25 °C for 24 h. After this period, the plates were analyzed using an inverted microscope, counting the total number of live larvae and dead larvae to calculate the survival percentage. The toxicity was evaluated based on the survival rates, considering the number of live larvae contained in a drop of positive larvae control compared with the treatment group. The experiment was performed in triplicate, and the results were expressed as mean ± standard deviation.

### 2.9. In Vivo Efficacy and Safety in Galleria mellonella

*Galleria mellonella* larvae were cultivated in a Biochemical Oxygen Demand (B.O.D.) at 27 °C until they reached a weight between 200 mg and 300 mg. The experimental groups consisted of 8 larvae (n = 8).

#### 2.9.1. Toxicity Evaluation

For toxicity studies, *G. mellonella* larvae (200–300 mg) were used following the specifications of Allegra et al. (2018) [28], with minor modifications. The larvae were fed a diet consisting of cornmeal, brewer’s yeast, soy flour, powdered milk, honey, and glycerol. Once the larvae reached the appropriate weight, they were separated, weighed, and kept away from light in an incubator at 25 °C without food supply for 24 h before the test.

The tested solutions were administered into the last left proleg of each larva after asepsis with 70% alcohol, using a 10 μL Hamilton Microliter^®^ syringe (Reno, NV, USA). The evaluated groups included ZPHA-VRC NPs (2, 10, and 50 mg/kg) and free VRC (2, 10, and 50 mg/kg). Control groups consisted of methanol 100% (mortality control), healthy larvae (health control), PBS buffer (nanoparticle diluent and injury control), 20% ethanol (VRC diluent control), and drug-free nanoparticles (ZPHA NPs).

After administration, the larvae were kept at 25 °C, and the toxic potential was assessed at intervals of 24, 48, 72, 96, and 120 h. Their health condition was evaluated based on the survival rate, melanization, cocoon formation, and activity (ability to move) [29]. Larvae were considered dead when they showed no reaction to physical stimulation. This experiment was performed in triplicate.

#### 2.9.2. Survival Curve After *C. albicans* Infection

To determine the appropriate concentration for assessing therapeutic efficacy, different inoculum concentrations (1 × 10^4^, 1 × 10^5^, 5 × 10^5^, and 1 × 10^6^ cells/larva) prepared in PBS buffer were tested. The experiment was conducted with 8 larvae per group (n = 8). One day before the test, the larvae were separated, weighed, and incubated at 37 °C without food.

A 10 μL inoculum was administered into the last left proleg of each larva after disinfection with 70% alcohol in a sterile laminar flow environment. The larvae were incubated at 37 °C for 5 days, and the survival rate was recorded daily. The experiment was performed in triplicate.

#### 2.9.3. Evaluation of Systemic Anti-*Candida albicans* Activity In Vivo

For systemic anti-*Candida albicans* activity, the reference isolate *C. albicans* ATCC 90028 was used. The strain was stored as frozen stock at −80 °C and subcultured onto Sabouraud Dextrose Agar (SDA) at 35 °C as needed.

Infection in *G. mellonella* was induced by administering 10 μL of inoculum (5 × 10^5^ cells/larva) into the left proleg of each larva, and the larvae were incubated for 2 h at 37 °C to allow infection development. Treatment was then administered by injecting 10 μL of ZPHA-VRC NPs (10 and 50 mg/kg) or free VRC (10 and 50 mg/kg) into the right proleg. Control groups included healthy larvae and untreated infected larvae. The larvae were incubated at 37 °C for 5 days, and evaluations were performed daily. Each group consisted of 12 larvae (n = 12). Health index parameters, including activity, cocoon formation, melanization, and survival, were assessed according to [29,30]. The experiment was replicated independently in triplicate.

Fungal burden was assessed 24 and 48 h post-treatment. Two larvae per group were selected, sanitized with 70% alcohol, decapitated, and homogenized in 1 mL of PBS buffer. The suspension was vortexed for 1 min, diluted 1:100 in PBS, and 100 μL of the diluted homogenate was plated on SDA supplemented with chloramphenicol. After 24 h of incubation at 37 °C, colony-forming units (CFUs) were counted to evaluate the therapeutic efficacy of the ZPHA-VRC NPs compared to the free VRC and control groups.

### 2.10. Statistical Analysis

Data were presented as mean ± standard deviation and analyzed using Minitab software (Version 22.2.1) for factorial design and GraphPad Prism 9 (Version 9.5.1,) for in vitro and in vivo experiments. Statistical significance was determined at *p* < 0.05.

## 3. Results and Discussion

### 3.1. Optimization of ZPHA-VRC NPs

**Figure 1:**
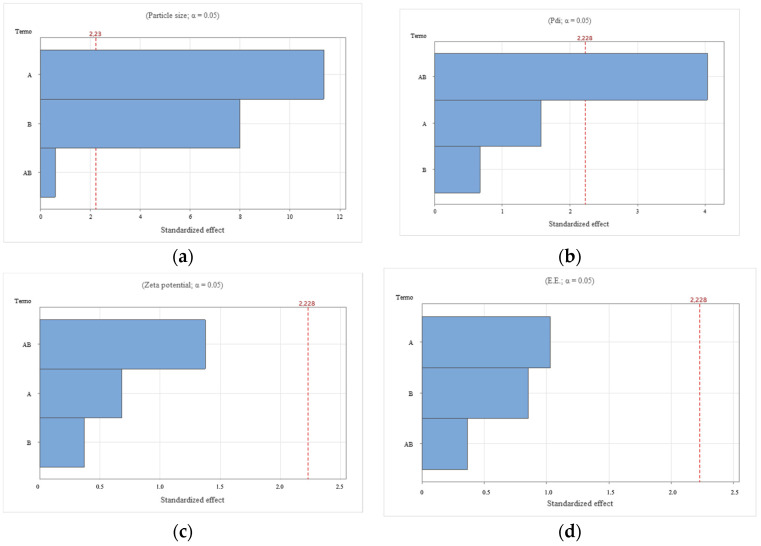
Pareto chart depicting the individual and interactive effects of zein and hyaluronic acid concentrations on key physicochemical properties of ZPHA-VRC nanoparticles: (**a**) particle size; (**b**) PDI; (**c**) zeta potential; and (**d**) entrapment efficiency (EE).

**Figure 2:**
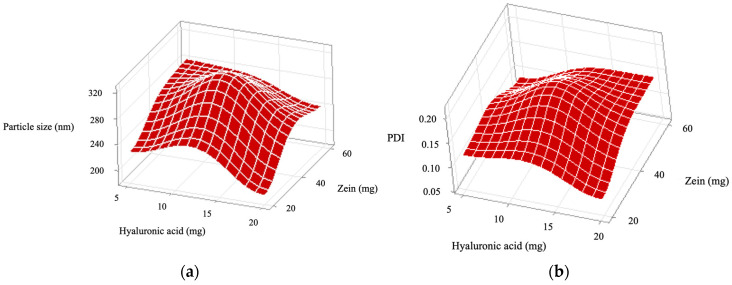

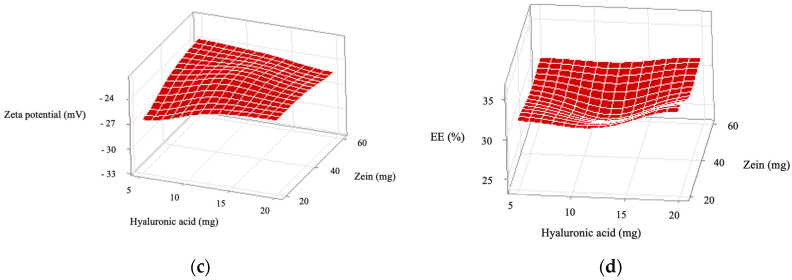
Response surface plots showing the individual and interactive effects of the independent variables, zein and hyaluronic acid concentrations, on the following properties of ZPHA-VRC nanoparticles: (**a**) particle size, (**b**) polydispersity index (PDI), (**c**) zeta potential, and (**d**) encapsulation efficiency (EE).

The particle size, PDI, zeta potential, and encapsulation efficiency of the prepared ZPHA-VRC NPs were systematically evaluated across various formulations (Table 2). These physicochemical properties were optimized and analyzed through statistical modeling (Figure 1) and response surface methodology (Figure 2). The results demonstrate how the formulation variables influenced the particle size, PDI, zeta potential, and EE, providing insights into the complex interplay between zein and hyaluronic acid concentrations.

The particle size of the nanoparticles ranged from 191.7 ± 6.1 nm (F2) to 319.3 ± 4.0 nm (F5). The particle size varied significantly with both the zein (X1) and hyaluronic acid (X2) concentrations, as evidenced by the Pareto chart (Figure 1a). Zein (F = 129.00, *p* < 0.0001) had the most significant influence, followed by hyaluronic acid (F = 63.85, *p* < 0.0001). The interaction (X1:X2) was not significant (F = 0.36, *p* = 0.561). The response surface plot for particle size (Figure 2a) shows a trend where higher zein concentrations resulted in larger particles, consistent with its role as a structural matrix. Conversely, increasing hyaluronic acid concentrations reduced the particle size, likely due to its contribution to the overall molecular organization and surface tension. The optimal combination, as observed in F2, produced the smallest particle size (191.7 ± 6.1 nm), which is favorable for nanoparticle stability and drug delivery.

PDI values reflect the uniformity of the particle size distribution, with lower values indicating better homogeneity. The PDI values, which provide insight into the uniformity of the nanoparticles, ranged from 0.079 ± 0.015 (F2) to 0.191 ± 0.024 (F5). The Pareto chart (Figure 1b) indicates that the interaction between zein and hyaluronic acid (X1:X2) was significant (F = 16.25, *p* = 0.002), while the main effects of zein (F = 2.49, *p* = 0.146) and hyaluronic acid (F = 0.45, *p* = 0.517) were not. This suggests that achieving uniformity depends on the precise balance between these components. The response surface plot (Figure 2b) confirms this interaction, highlighting areas of low PDI at specific zein and hyaluronic acid concentrations. F2 and F4, with PDI values of 0.079 ± 0.015 and 0.089 ± 0.005, respectively, exemplify formulations with excellent size uniformity.

The zeta potential was consistently negative across all formulations, ranging from −26.6 ± 5.1 mV (F1) to −23.3 ± 1.0 mV (F5), indicating good colloidal stability. Statistical analysis revealed no significant effects of zein (F = 0.47, *p* = 0.510), hyaluronic acid (F = 0.14, *p* = 0.717), or their interaction (F = 1.89, *p* = 0.199). The Pareto chart (Figure 1c) and response surface plot (Figure 2c) support this finding, showing minimal variation in the zeta potential across the experimental range. These results confirm that the negative charge primarily stems from hyaluronic acid, ensuring electrostatic repulsion and preventing aggregation.

EE ranged from 28.4 ± 4.2% (F5) to 33.8 ± 2.2% (F2), with no significant effects observed for zein (F = 1.07, *p* = 0.326), hyaluronic acid (F = 0.73, *p* = 0.412), or their interaction (F = 0.13, *p* = 0.721). The Pareto chart (Figure 1d) and the response surface plot (Figure 2d) show a relatively flat profile, indicating that EE is less sensitive to changes in formulation parameters.

F2 was chosen for the subsequent in vitro and in vivo experiments. The Pareto and response surface plots provide visual confirmation of the statistical findings, elucidating the critical effects of zein and hyaluronic acid concentrations on the nanoparticle size and PDI. These properties showed strong dependence on both the main effects and interaction, whereas the zeta potential and EE were less influenced. These observations underscore the importance of carefully optimizing formulation parameters to achieve nanoparticles with desirable characteristics for drug delivery applications.

### 3.2. Determination of Antifungal Activity In Vitro

The MIC values of fluconazole, free VRC, ZPHA-VRC NPs, and blank zein–hyaluronic acid nanoparticles (ZPHA NPs) were evaluated against *Candida* species, including *C. albicans* ATCC 90028, *C. krusei* ATCC 6258, and *C. parapsilosis* ATCC 22019. The results are summarized in Table 3.

VRC exhibited the highest antifungal efficacy across all tested *Candida* species, demonstrating its potent activity against both fluconazole-sensitive and fluconazole-resistant *Candida* strains. The superior performance of VRC to fluconazole aligns with its broader spectrum of activity and higher potency, particularly against fluconazole-resistant species such as *C. krusei* [7]. However, we cannot state that the performance of free VRC was superior in relation to ZPHA-VRC NPs since the concentrations do not differ by more than 2× between the samples. The MIC values obtained in this study align with previously reported data for voriconazole against *Candida* species. The literature data indicate that voriconazole exhibits MIC values typically ranging from 0.008 to 0.03 µg/mL for *C. albicansand C. parapsilosis*, while *C. krusei*, known for its intrinsic resistance mechanisms, presents MIC values between 0.125 and 0.5 µg/mL [31]. The MIC values determined in this study fall within these established ranges, confirming the antifungal potency of voriconazole in both its free and nanoparticle-encapsulated forms.

Species-specific susceptibility patterns were observed, with *C. albicans* and *C. parapsilosis* being more sensitive to both free VRC and ZPHA-VRC NPs. In contrast, *C. krusei* demonstrated reduced susceptibility, reflecting its inherent resistance mechanisms to azole antifungals. This trend was consistent for both the free drug and the encapsulated formulation, indicating that the nanoparticle delivery system does not significantly alter the species-specific activity of VRC.

Blank ZPHA NPs exhibited no antifungal activity against any of the tested species, confirming that the antifungal effects of the ZPHA-VRC formulation were entirely due to the presence of VRC. This finding underscores the biocompatibility of the nanoparticle system, as it does not interfere with fungal growth in the absence of the drug.

The slightly reduced activity of ZPHA-VRC NPs compared to free VRC is an expected trade-off for the benefits provided by the nanoparticle system, such as controlled drug release, potential reduction in systemic toxicity, and improved bioavailability. These properties could be advantageous in clinical settings, particularly for the management of chronic fungal infections requiring sustained drug release and reduced side effects.

### 3.3. Cytotoxicity Evaluation on Vero Cells

**Figure 3:**
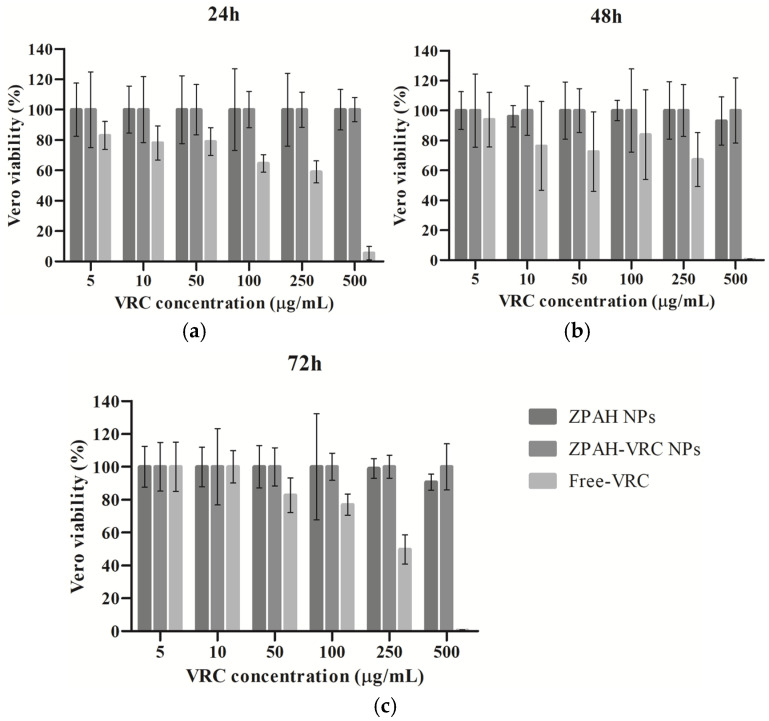
Inhibition rates of Vero cells treated with ZHA-VRC NPs, ZHA NPs, and free VRC after (**a**) 24 h, (**b**) 48 h, and (**c**) 72 h of exposure.

The cytotoxicity of ZPHA-VRC NPs, blank nanoparticles (ZPHA NPs), and free VRC was assessed on Vero cells at various concentrations (5 to 500 µg/mL) over 24, 48, and 72 h (Figure 3). The percentage of cell viability was measured to determine the safety profile of the formulations compared to the free drug.

The results revealed that both ZPHA NPs and ZPHA-VRC NPs exhibited minimal cytotoxicity across all tested concentrations and exposure times. Cell viability remained above 80% for both nanoparticle formulations, even at the highest concentration of 500 µg/mL. These findings suggest that the nanoparticle carrier system, composed of zein and hyaluronic acid, is highly biocompatible and does not induce significant cytotoxic effects on Vero cells. Similarly, other studies have reported that zein nanoparticles exhibit minimal cytotoxicity in Vero cells, further supporting their biocompatibility and suitability for drug delivery applications [32,33].

In contrast, free VRC displayed a concentration-dependent decrease in cell viability, particularly at the highest concentration (500 µg/mL), where viability dropped significantly below 50%. This result highlights the potential cytotoxicity of free VRC at elevated concentrations, which could limit its clinical applicability in high-dose scenarios. The encapsulation of VRC within ZPHA nanoparticles reduces this cytotoxicity, likely due to the controlled release mechanism of the nanoparticle system, which reduces the immediate exposure of cells to high concentrations of the drug.

No significant differences in cell viability were observed between the 24, 48, and 72 h time points for ZPHA NPs and ZPHA-VRC NPs, indicating that the nanoparticle formulations maintain a stable and consistent safety profile over time. For free VRC, the concentration-dependent cytotoxicity was consistent across the different time points, further emphasizing the advantages of the nanoparticle-based delivery system in reducing cellular toxicity.

The markedly lower cytotoxicity of ZPHA-VRC NPs compared to free VRC underscores the potential of nanoparticle-based delivery systems to improve the safety profile of antifungal therapies. This biocompatibility is particularly critical for applications involving prolonged treatment regimens or high drug doses, where systemic and cellular toxicity are major concerns.

### 3.4. In Vivo Toxicity in the Caenorhabditis elegans Model

**Figure 4:**
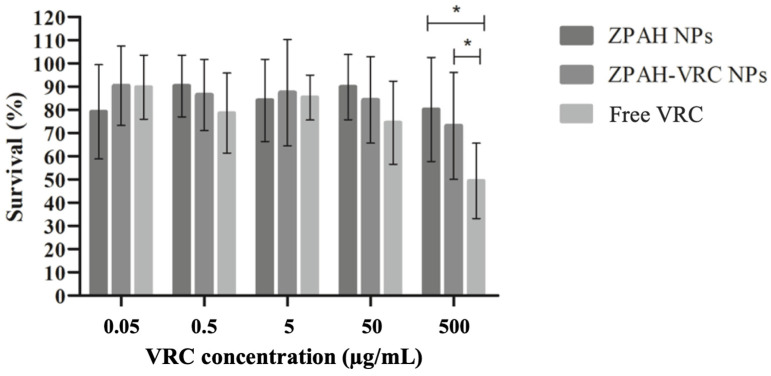
Survival rates of *C. elegans* exposed to different concentrations of ZPAH-VRC NPs, ZPAH NPs, and free VRC. * *p* < 0.05.

The survival of C. elegans was evaluated following exposure to ZPHA-VRC NPs, ZPHA NPs, and free VRC across a range of concentrations (0.05 to 500 µg/mL). The results, depicted in Figure 4, provide important insights into the safety profile of the formulations.

The survival rates of *C. elegans* remained high (>80%) for ZPHA NPs and ZPHA-VRC NPs at all tested concentrations, indicating that the nanoparticle systems are biocompatible and do not induce significant toxicity to the nematodes. Even at the highest concentration of 500 µg/mL, no substantial reduction in survival was observed, suggesting that the nanoparticle formulations are well-tolerated.

In contrast, exposure to free VRC resulted in a significant, concentration-dependent decrease in *C. elegans* survival. The most pronounced effect was observed at 500 µg/mL, where survival rates dropped substantially, highlighting the potential toxicity of free VRC at elevated concentrations. These findings align with the results from the cytotoxicity assay in Vero cells, further emphasizing the advantages of encapsulating VRC in a nanoparticle system to mitigate its inherent cytotoxic effects. Similarly, Lucio et al. (2017) [34] demonstrated that zein nanoparticles encapsulating glibenclamide were non-toxic to *Caenorhabditis elegans*, with no significant changes in worm viability after 48 h of exposure, aligning with our findings that ZHA NPs and ZHA-VRC NPs also exhibited no toxicity in this alternative nematode model.

The high survival rates of *C. elegans* exposed to ZPHA-VRC NPs and ZPHA NPs underscore their potential for safe application in biological systems. The findings suggest that these nanoparticle formulations could be used to deliver VRC in a controlled and biocompatible manner, reducing the risk of systemic toxicity. The results also highlight the value of *C. elegans* as a model organism for preliminary toxicity evaluations, offering a simple and cost-effective alternative to mammalian models.

### 3.5. In Vivo Efficacy and Safety in G. mellonella

#### 3.5.1. Toxicity

**Figure 5:**
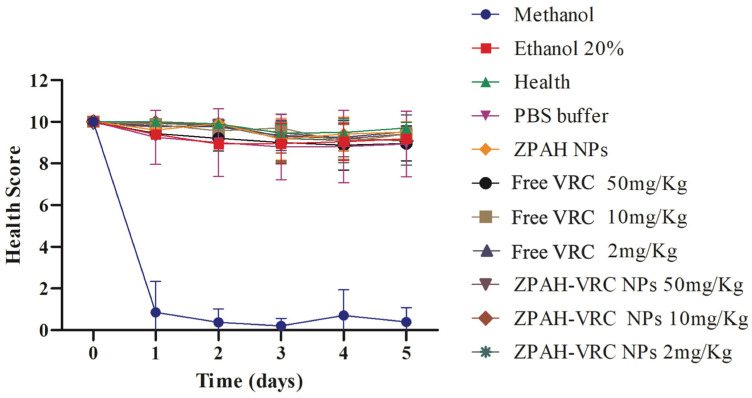
Health scores of *G. mellonella* larvae over five days following exposure to ZPAH-VRC NPs, ZPAH NPs, free VRC, and control substances. The results are expressed as mean ± standard deviation. Two-way ANOVA followed by Friedman.

The in vivo toxicity of ZPHA-VRC NPs, blank nanoparticles (ZPHA NPs), and free VRC was evaluated using the *G. mellonella* model as an alternative system to assess drug safety. The health scores of the larvae were monitored over five days following treatment with various formulations and dosages, and the results revealed significant differences in toxicity profiles (Figure 5).

The control groups treated with methanol exhibited rapid and severe toxicity, as indicated by a sharp decline in the health scores to nearly zero by the second day of observation. These findings highlight the toxic nature of these solvents to *G. mellonella*. Conversely, larvae treated with PBS, the healthy untreated group, and those treated with ZPHA NPs maintained consistently high health scores throughout the experimental period, confirming the biocompatibility of the nanoparticle carrier and the inert nature of the other control substances.

Free VRC displayed a dose-dependent toxicity profile. The larvae exposed to the highest dose of 50 mg/kg exhibited a marked decline in health scores, suggesting significant systemic toxicity at elevated concentrations. At lower doses (10 mg/kg and 2 mg/kg), the larvae demonstrated improved survival, maintaining moderate to high health scores. These results indicate that although VRC is an effective antifungal agent, its use at high doses may pose risks of systemic toxicity, which could limit its clinical application.

In contrast, the ZPHA-VRC nanoparticles exhibited a markedly improved safety profile. The larvae treated with ZPHA-VRC nanoparticles at all tested doses (2, 10, and 50 mg/kg) maintained consistently high health scores comparable to those of the healthy untreated and PBS control groups. This indicates that encapsulating VRC within ZPHA nanoparticles effectively mitigates the drug’s inherent toxicity. The controlled release properties of the nanoparticles likely contribute to this reduced toxicity by lowering the peak systemic concentrations of the drug, thereby minimizing its adverse effects on the larvae.

The distinct toxicity profiles between free VRC and ZPHA-VRC NPs underscore the potential of nanoparticle-based delivery systems to enhance the safety of antifungal therapies. Encapsulation within the biocompatible zein–hyaluronic acid matrix provides a protective mechanism that reduces the immediate exposure of biological systems to high drug concentrations, ensuring a safer therapeutic approach. The results also demonstrate the utility of the *G. mellonella* model in assessing the in vivo safety of drug formulations, offering a rapid and cost-effective alternative to mammalian models.

#### 3.5.2. Antifungal Efficacy

**Figure 6:**
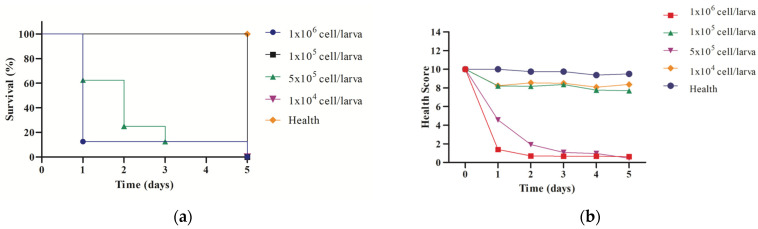
Preliminary analysis to evaluate the survival curve (**a**) and the health scores of (**b**) the *G. mellonella* larvae in response to different inoculum concentrations of *C. albicans*.

The doses of 10 mg/kg and 50 mg/kg were selected for the therapeutic efficacy study in a Candida albicans infection model using *G. mellonella*. These concentrations were chosen based on the toxicity studies, which showed no significant harmful effects for any of the tested substances. Before initiating the therapeutic efficacy study, a preliminary analysis was conducted to evaluate the survival curve and the health scores of the larvae in response to different inoculum concentrations of *C. albicans* (Figure 6). The goal was to identify an inoculum concentration capable of inducing infection without causing accelerated mortality.

The survival analysis (Figure 6a) revealed a clear dose-dependent relationship between C. albicans inoculum concentration and larval survival. Larvae infected with the highest concentration (1 × 10^6^ cells/larva) exhibited a rapid decline in survival, with all larvae succumbing to infection within two days. In contrast, larvae infected with the lowest concentration (1 × 10^4^ cells/larva) maintained high survival rates comparable to the healthy control group. Intermediate inoculum concentrations (5 × 10^5^ and 1 × 10^5^ cells/larva) showed a progressive decline in survival, with 5 × 10^5^ cells/larva resulting in a survival rate of approximately 20% by day 3. Based on these findings, the inoculum concentration of 5 × 10^5^ cells/larva was selected for the therapeutic efficacy study, as it effectively established infection without causing excessively rapid mortality.

In addition to survival, the health scores of the larvae (Figure 6b) were evaluated to monitor the progression of infection across different inoculum concentrations. The larvae infected with 1 × 10^6^ cells/larva exhibited a rapid and significant decline in health scores within the first 24 h, confirming the severity of the infection at this concentration. For the 5 × 10^5^ cells/larva group, the health scores decreased more gradually over time, providing a window for therapeutic intervention. At lower concentrations (1 × 10^5^ and 1 × 10^4^ cells/larva), the health scores remained relatively stable, particularly in the 1 × 10^4^ cells/larva group, where the scores closely resembled those of the healthy control group.

The results of this preliminary study demonstrate that the inoculum concentration of 5 × 10^5^ cells/larva strikes a balance between establishing a reproducible infection model and maintaining a timeframe that allows for meaningful evaluation of therapeutic interventions. The ability of this model to differentiate infection severity based on health scores and survival rates highlights its utility in evaluating antifungal efficacy in vivo.

**Figure 7:**
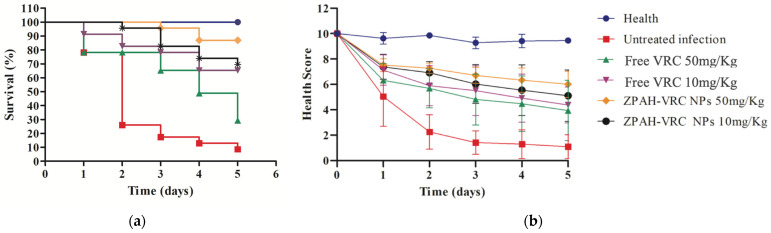
Therapeutic efficacy in *G. mellonella* larvae infected with *C. albicans* ATCC 90028 (5 *×* 10^5^ cells/worm) and treated with ZHA-VRC NPs and free VRC over five days: (**a**) survival analysis; (**b**) health scores.

The therapeutic efficacy of ZPHA-VRC NPs was evaluated in a *C. albicans* infection model using *G. mellonella*. The survival and health scores of the larvae were monitored over five days and compared across groups treated with ZPHA-VRC NPs (10 mg/kg and 50 mg/kg), free VRC (10 mg/kg and 50 mg/kg), an untreated infected group, and a healthy control group. The results are shown in Figure 7.

The survival curves (Figure 7a) demonstrated significant differences between the treated and untreated groups. The larvae in the untreated infected group experienced rapid mortality, with less than 20% survival by day 3 and complete mortality by day 5. This sharp decline in survival reflects the severity of the fungal infection in the absence of therapeutic intervention. In contrast, all treatment groups showed improved survival rates. Among these, ZPHA-VRC NPs at both tested doses provided superior protection, with survival rates of approximately 80% (50 mg/kg) and 70% (10 mg/kg) by day 5. The free VRC treatments were less effective, with survival rates of 60% (50 mg/kg) and 50% (10 mg/kg), highlighting the enhanced therapeutic efficacy of the nanoparticle-based delivery system.

The health scores (Figure 7b) further supported the findings from the survival analysis. The untreated infected group exhibited a rapid and significant decline in health scores, reaching near-zero levels by day 3. The larvae treated with free VRC demonstrated a dose-dependent effect, with higher health scores observed for the 50 mg/kg dose compared to the 10 mg/kg dose. However, the health scores for both doses of ZPHA-VRC NPs were consistently higher than those of the free VRC groups throughout this study. The nanoparticle-treated groups maintained health scores close to 8–9 on day 5, indicating a less severe infection progression and improved overall health compared to the free drug-treated groups.

**Figure 8:**
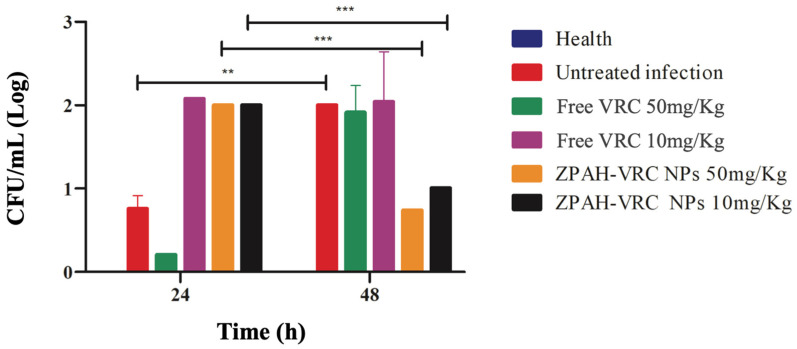
Fungal burden reduction (CFU/mL) in *G. mellonella* larvae infected by *C. albicans* ATCC 90028 (5 × 10^5^ cells/worm) after treatment with ZHA-VRC NPs and free VRC over 24 and 48 h. Statistical analysis was performed using two-way ANOVA followed by Sidak’s post hoc test. Asterisks (** and ***) indicate statistically significant differences between 24 and 48 h (** *p* < 0.001, *** *p* < 0.0001).

The reduction in fungal burden in *G. mellonella* larvae was evaluated at 24 and 48 h post-infection to further assess the therapeutic efficacy of ZHA-VRC NPs compared to free VRC and the untreated infection group. Colony-forming units (CFUs) were quantified, and the results are shown in Figure 8.

The fungal burden analysis provided direct evidence of the therapeutic efficacy of the formulations. At 24 h post-infection, all treated groups showed significant reductions in fungal burden compared to the untreated group, with ZHA-VRC NPs at 50 mg/kg achieving the greatest reduction. By 48 h, the fungal burden in the untreated group had markedly increased, reflecting rapid fungal proliferation. The free VRC treatment moderately reduced fungal burden but was less effective than the nanoparticle formulation. Notably, ZHA-VRC NPs at 50 mg/kg nearly suppressed fungal growth, while the 10 mg/kg nanoparticle group also maintained a significant reduction compared to free VRC at equivalent doses.

The combined findings from survival, health score, and fungal burden assessments demonstrate the superior therapeutic efficacy of ZPHA-VRC NPs compared to free voriconazole, an advantage for clinical applications. The nanoparticles significantly improved survival rates in infected *G. mellonella*, reduced fungal burden, and maintained higher health scores, indicating enhanced treatment outcomes. The improved safety profile observed in both *C. elegans* and *G. mellonella* models suggests a potential reduction in systemic toxicity, which could be clinically beneficial in mitigating voriconazole-associated adverse effects. These results highlight the potential of ZPHA-VRC NPs as a promising alternative for antifungal therapy, particularly for patients requiring prolonged treatment, where toxicity is a major concern. The use of nanoparticle-based delivery systems may also improve drug stability and distribution, enhancing treatment efficacy in systemic fungal infections. Furthermore, the *G. mellonella* model proved to be a valuable tool for evaluating antifungal efficacy and drug delivery systems, providing a cost-effective, reliable, and reproducible platform [35,36]. However, their clinical applicability depends on a deeper evaluation of critical factors, such as the scalability of the synthesis process and the challenges associated with transitioning to human models. Large-scale production may face barriers related to reproducibility, stability, and manufacturing costs, requiring optimizations to ensure commercial viability. Furthermore, the extrapolation of in vitro and alternative model data to clinical settings necessitates studies that account for more complex biological interactions, including biodistribution, metabolism, and potential adverse effects. While the findings of this research represent an advancement in nanotechnology applied to antifungal therapy, future investigations are essential to validate its efficacy and safety in real clinical contexts.

## 4. Conclusions

This study successfully developed and optimized ZPHA-VRC NPs, presenting a promising strategy for antifungal therapy. The nanoparticles demonstrated favorable physicochemical properties, reduced cytotoxicity, enhanced the safety profile of VRC, and maintained robust antifungal activity against *Candida* species. Alternative models, such as *G. mellonella* and *C. elegans*, were instrumental in evaluating the safety and efficacy of the formulations. These models provided ethical and cost-effective platforms for assessing toxicity and therapeutic potential, offering insights that bridge the gap between in vitro studies and mammalian models. The *G. mellonella* infection model, in particular, demonstrated the superior therapeutic efficacy of ZPHA-VRC NPs compared to free VRC. In summary, ZPHA-VRC NPs offer a safer and more effective delivery system for VRC, with significant potential to improve antifungal therapy. The integration of alternative efficacy and safety models highlights their value in preclinical research, supporting the advancement of nanoparticle-based therapeutics toward clinical applications.

## Figures and Tables

**Table 1:** Coded and real variables for the 2^2^-factorial design of ZPHA-VRC NPs.

Experiment	X1 (Coded)	X2 (Coded)	Zein (mg)	Hyaluronic Acid (mg)
F1	+1	+1	20	5
F2	+1	−1	20	20
F3	−1	−1	60	20
F4	−1	+1	60	5
F5	0	0	40	12.5

Each experiment consisted of the preparation of independent formulations in triplicate.

**Table 2:** Physicochemical responses of ZPHA-VRC NPs based on a 2**^2^**-factorial design: particle size, polydispersity index (PDI), zeta potential, and encapsulation efficiency (EE).

Experiment	Particle Size (nm)	PDI	Zeta Potential (mV)	EE (%)
F1	225.9 ± 4.7 ^c^	0.117 ± 0.018 ^b,c^	−26.6 ± 5.1 ^a^	31.8 ± 3.1 ^a^
F2	191.7 ± 6.1 ^d^	0.079 ± 0.015 ^c^	−24.1 ± 0.7 ^a^	33.8 ± 2.2 ^a^
F3	241.5 ± 5.8 ^c^	0.143 ± 0.027 ^a,b^	−25.1 ± 1.1 ^a^	31.5 ± 2.1 ^a^
F4	281.3 ± 4.0 ^b^	0.089 ± 0.005 ^c^	−23.7 ± 1.1 ^a^	30.7 ± 1.2 ^a^
F5	319.3 ± 4.0 ^a^	0.191 ± 0.024 ^a^	−23.3 ± 1.0 ^a^	28.4 ± 4.2 ^a^

Results are expressed as mean ± standard deviation (n = 3). Same letters mean statistical equality and different letters statistical inequality, analyzed by column. One-way ANOVA, followed by Tukey’s test, *p* < 0.05.

**Table 3:** Minimum inhibitory concentrations (MICs) of FLU, VRC, ZHA-VRC NPs, and ZHA NPs against *Candida* species.

Species		FLU (µg/mL)	VRC (µg/mL)	ZPHA-VRC NPs (µg/mL)	ZPHA NPs (µg/mL)
	MIC				
*C. albicans* ATCC 90028		2	0.015	0.030	No activity
*C. krusei* ATCC 6258		32	0.250	0.125	No activity
*C. parapsilosis* ATCC 22019		2	0.015	0.030	No activity

MIC: minimum inhibitory concentration; FLU: fluconazole; VRC: voriconazole; ZHA-VRC NPs: nanoparticle with voriconazole; ZHA NPs: nanoparticle without voriconazole.

## Data Availability

Data will be made available upon request.

## Abbreviations

The following abbreviations are used in this manuscript:

VCR	voriconazole
ZPHA-VRC NPs	zein–pectin–hyaluronic acid nanoparticles containing voriconazole
ZPHA-NPs	zein–pectin–hyaluronic acid nanoparticles without voriconazole
